# Isolation and multilocus sequence typing of *Borrelia burgdorferi* from *Ixodes scapularis* collected from dogs in Ontario, Canada

**DOI:** 10.1186/s13104-023-06315-0

**Published:** 2023-03-30

**Authors:** Grace K. Nichol, J. Scott Weese, Katie M. Clow

**Affiliations:** 1 Department of Population Medicine, Ontario Veterinary College, University of Guelph, 50 Stone Road East, Guelph, ON N1G 2W1 Canada; 2 Department of Pathobiology & the Centre for Public Health and Zoonoses, Ontario Veterinary College, University of Guelph, 50 Stone Road East, Guelph, ON N1G 2W1 Canada

**Keywords:** *Borrelia burgdorferi*, *Ixodes scapularis*, Multilocus sequence typing

## Abstract

**Objective:**

To identify the multilocus sequence typing (MLST) sequence types of *Borrelia burgdorferi* from *Ixodes scapularis* in Ontario, Canada.

**Results:**

One hundred and eighty-five *I. scapularis* ticks were submitted from 134 dogs via participating clinics from April 1, 2019, to March 31, 2020. Seventeen MLST sequence types of *B. burgdorferi* were detected from fifty-eight cultured isolates from 21 ticks. The most common MLST sequence types were 12 and 16. Mixed infections of two MLST sequence types were detected in four ticks. Three sequence types (48, 317, 639) were new detections in Ontario.

## Introduction

Over the last two decades, there has been rapid northward range expansion of the blacklegged tick, *Ixodes scapularis*, in Ontario, Canada [1, 2]. This tick is the vector for several pathogens, including *Borrelia burgdorferi* sensu stricto (herein referred to as *B. burgdorferi*), the causative agent of Lyme disease in humans, dogs, and horses [3–5]. Lyme disease is the most common human vector-borne disease in Canada, with a notable increase in case counts from 144 reported in 2009 to 3147 reported in 2021 [6]. Evidence of exposure to *B. burgdorferi* in dogs and horses has also been detected in areas of central and eastern Canada [7, 8].

*Borrelia burgdorferi* sensu stricto can be further characterized by genetic analyses. Restriction fragment length polymorphism (RFLP) analysis of the 16-23 S intragenic spacer (IGS) region and sequencing of the plasmid-encoded OspA and OspC surface proteins have been employed [9–11]. Multilocus sequence typing (MLST) uses the combined sequences of eight chromosomal bacterial housekeeping genes. Given these housekeeping genes are slow to evolve, this approach has been shown to have higher discriminatory power than previously utilized methods to characterize genetic diversity and is currently the recommended approach [9–11].

Associations have been detected between sequence types of *B. burgdorferi* and geographic distribution, reservoir host species, immune response in humans and severity of human clinical disease [12–19]. Approximately 5% of dogs exposed to *B. burgdorferi* will develop clinical disease, which is characterized by fever, anorexia and shifting lameness, and in rare cases, the disease can progress to a potentially fatal protein-losing nephropathy [4]. Similarly in horses, only a subset of those exposed develop clinical disease, with three documented syndromes: cutaneous pseudolymphoma, uveitis, and a highly fatal neuroborreliosis [5]. It is unknown if the variability of clinical disease presentation and severity in dogs and horses has any association with *B. burgdorferi* sequence type.

As emergence of *I. scapularis* and *B. burgdorferi* continue across Ontario, it is pertinent to expand our knowledge base on sequence types and the associated ecological, epidemiological, and clinical patterns. To date, a handful of studies have characterized MLST sequence types of *B. burgdorferi* in Canada [12–17]. This study builds on these efforts by describing the MLST sequence types of *B. burgdorferi* in ticks collected from dogs in southern Ontario.

## Methods

A convenience sample of veterinary clinics from across Ontario was invited to participate in this study via email. Live *I. scapularis* ticks were collected from dogs (e.g., the tick could be attached or crawling on the animal) at participating clinics and shipped immediately to our laboratory during a one-year period (April 2019 – March 2020). Each tick submission was accompanied by a questionnaire that documented patient information, including use of antibiotics during the last two months, suspected geographic location of tick acquisition, and date of tick removal.

Tick samples were morphologically identified using a stereoscope and standard keys to confirm the species identification, life stage, and sex (for adult samples) [20]. Ticks were surface disinfected by submerging and briefly vortexing each sample sequentially in the following reagents: sterile water, 0.5 − 1% sodium hypochlorite, 0.5% benzalkonium chloride, 3% hydrogen peroxide, 70% ethanol, sterile water. Each tick was macerated using a sterile scalpel blade, and a tick homogenate was prepared by adding 969 µl 1.5x complete BSK and 31 µl of an antibiotic mixture (phosphomycin (2 mg/mL), rifampicin (5 mg/mL), amphotericin B (0.25 mg/mL), kanamycin (10 µg/mL)). At this point, a 100 µL aliquot was removed from the sample for DNA extraction followed by real-time PCR targeting the 23S rRNA gene of *B. burgdorferi* sensu lato, as previously described [21]. DNA was extracted using the DNeasy Blood and Tissue Kit [Qiagen, Toronto, ON] per manufacturer’s protocol, except ticks were incubated with the digestive reagent, proteinase K, overnight (vs. the prescribed 2 h). Real-time PCR runs included both negative (water) and positive controls. The tick homogenates of samples that were PCR positive for *B. burgdorferi* were plated using a reagent combination of 20 mL 1.5x complete BSK, 10 mL 2.1% agarose, and 930 µL antibiotic mixture. Plates were placed in a 37^o^ Celsius incubator with 5% CO_2_ and monitored for at least 31 days. If colonies appeared, three colonies were sampled from each plate to prepare isolates. In cases where there were less than three colonies present, all colonies were taken. Each colony was extracted by removing the agarose plug and placing it in BSK-H with 10% glycerol for expansion. A 150 µL aliquot was removed for DNA extraction (as described above), followed by multilocus sequence typing (MLST). MLST was conducted for eight housekeeping genes (*clpA, clpX, nifS, pepX, pyrG, recG, rplB* and *uvrA*). Bidirectional automated Sanger sequencing was performed using previously published primers [9, 10]. Forward and reverse reads were assembled and trimmed using Geneious Prime (Version 2020.2.4, Biomatters Ltd., Auckland, New Zealand, https://www.geneious.com). Chromatograms were visualized to validate base calls. Sequences were then uploaded to PubMLST to determine locus identities and sequence types [22].

Spatial data were prepared using QGIS version 3.22.13 (www.qgis.org; 2022). Vector layers were accessed through the Scholars GeoPortal at the University of Guelph (http://geo2.scholarsportal.info).

## Results

**Fig. 1:**
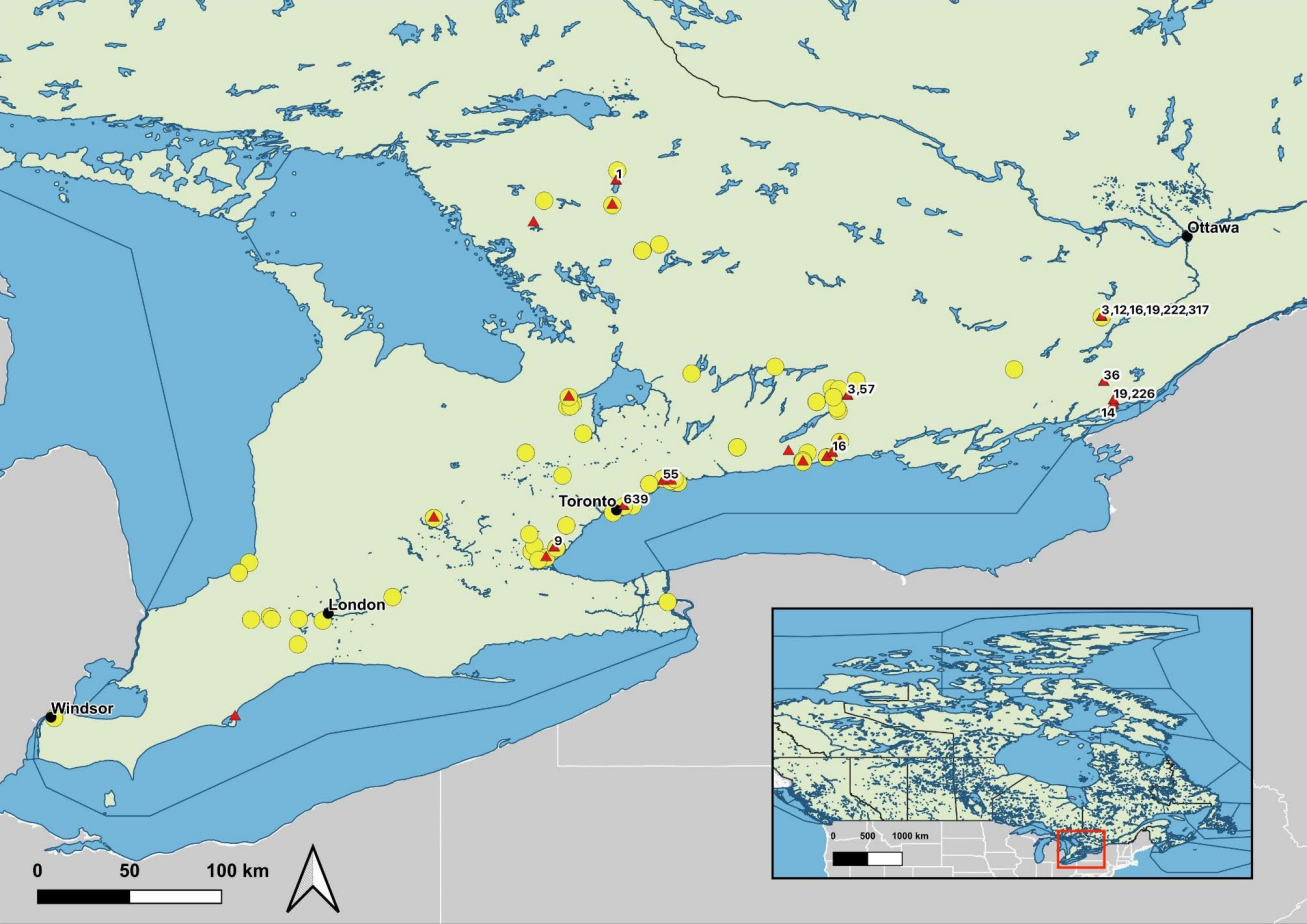
*Ixodes scapularis* removed from dogs were received from locations across southern Ontario and tested via real-time PCR for *B. burgdorferi* (positive = red triangle, negative = yellow circle). Positive samples were further analyzed to determine MLST sequence type (labels associated with red triangles). Longitude and latitude of tick location were based on owner-reported suspected location of tick acquisition. If a location was described but it was not specific (i.e., ‘backyard’), the tick was geocoded to the submitting veterinary clinic. Samples for which the location of tick acquisition could not be determined (e.g., missing data, numerous locations listed) have not been visually projected

One hundred and eighty-five *I. scapularis* ticks were submitted from 134 dogs from 19 participating clinics from April 1, 2019, to March 31, 2020 (Fig. 1). Nineteen of these ticks (10.3%) were submitted from 12 dogs that either had reported antibiotic use (5 dogs, 3.7%) or an unknown history (i.e., no information was provided, or owner did not know) (7 dogs, 5.2%). Thirty-eight (20.5%) ticks from 29 (21.6%) dogs were positive for *B. burgdorferi* by PCR. Four of these positive ticks were from dogs with reported or unknown antibiotic use. *Borrelia burgdorferi* was isolated from 21/38 (55.2%) PCR positive ticks. No culture growth was detected from the four positive ticks removed from dogs with reported or unknown antibiotic use. Fifty-eight *B. burgdorferi* isolates were subjected to MLST, but read quality was poor for three isolates. In total, 55 isolates were successfully typed. Seventeen *B. burgdorferi* MLST sequence types were detected, all of which have been previously described (Table 1). Four ticks had mixed infections, with isolates of different MLST sequence types identified concurrently (Table 1).

**Table 1:** *Borrelia burgdorferi* MSLT sequence types identified from ticks removed from dogs in Ontario, Canada

Tick sample	Isolate 1 sequence type	Isolate 2 sequence type	Isolate 3 sequence type	Location (latitude, longitude)
A	16	16	16	44.02, -77.99
B	34	34	34	Unknown^c^
C	16	16	16	Unknown^c^
D	55	55	55	43.83, -79.08
E	16	16	16	44.90, -76.25
F	222	12	12	44.90, -76.25
G	48	48	48	44.90, -76.25
H	12	16	12	44.90, -76.25
I	317	12	317	44.90, -76.25
J	12	12	12	44.90, -76.25
K	14	14	14	44.33, -76.17
L	226	19	226	44.36, -76.17
M	639	639	639	43.68, -79.34
N	3	N/A^a^	N/A^a^	44.90, -76.25
O	19	19	N/A^a^	44.90, -76.25
P	57	57	57	44.39, -77.90
Q	3	3	3	44.39, -77.90
R	18	Unknown^b^	18	Unknown^c^
S	36	N/A^a^	N/A^a^	44.48, -76.24
T	1	1	1	45.78, -79.38
U	9	Unknown^b^	Unknown^b^	43.41, -79.79

^a^Not applicable as fewer than three colonies grew for that sample.

^b^Sequence data of insufficient quality (e.g., truncated, inability to call several base pairs across multiple loci).

^b^The location of tick acquisition was unable to be determined due to insufficient information provided with the sample.

## Discussion

A diversity of *B. burgdorferi* MLST sequence types were isolated from *I. scapularis* collected in Ontario, Canada. Fourteen of the MLST sequence types isolated in this study have widespread distribution across northeastern and/or midwestern North America and have been previously identified from ticks and/or hosts in Ontario [12–15, 17]. However, three *B. burgdorferi* MLST sequence types identified in our study had only been reported in the provinces of Manitoba (48, 639) and Prince Edward Island (317), and thus represent new records for Ontario [14, 22]. Tick host movement, particularly that of migratory birds, has driven long distance dispersal of *I. scapularis*. However, this is predominately along a north-south corridor and these provinces are to the west (Manitoba) and east (Prince Edward Island) of Ontario [23]. Movement of reservoir hosts, such as small mammals, via connected habitats, has been associated with geographic patterns of MLST sequence types [17]. In this context, movement occurs on a local scale, not vast distances between these provinces. Additional research is needed to elucidate the ecological processes that drive MSLT sequence type diversity and dispersal. Moreover, as we develop a comprehensive understanding of geographic distribution of sequence types, further studies are warranted to explore the relevance of these sequence types to the epidemiology and clinical manifestation of Lyme disease, not only in humans, but dogs and horses as well.

### Limitations

The main limitations for this study were:

- Specimen quality: To detect and subsequently culture *B. burgdorferi* from *I. scapularis*, high quality live specimens are required. Although collecting ticks via veterinary clinics was an efficient way to acquire samples, many were highly engorged, and this can impact DNA extraction and thus PCR analyses. Although all samples were requested to be shipped immediately, some ticks were dead upon arrival, which may have contributed to unsuccessful attempts to culture *B. burgdorferi*.
- Geographic data: Location of tick acquisition was acquired through an owner-reported survey. Ticks can be attached for several days, over which time a dog can visit multiple locations, so it can be difficult to determine the precise location at which the tick was acquired.
- Sample size: Given only a small number of isolates were available for MLST, there was insufficient power to explore any advanced spatial analyses (e.g., cluster analysis).
- Genetic analysis: Although MLST has high discriminatory power, additional insight could have been gained by conducting whole genome analysis of isolates.

## Data Availability

*Borrelia* spp. isolates from this study have been uploaded to PubMLST with identification numbers 3456 to 3510 (https://pubmlst.org).
